# Dental Legislation

**Published:** 1899-04

**Authors:** G. H. Wilson

**Affiliations:** Cleveland, Ohio


### Dental Legislation.

BY G. H. WILSON, D.D.S., CLEVELAND, OHIO.

A talk given at the State Dental Society, December, 1898.

This is a subject of great interest to all the dentists of the State and throughout our whole country. Ohio should take an active part in this work at the present time. If you will think over the history of dental legislation, you will remember that Ohio was a leader in this branch of work. Alabama introduced the first dental law, but it was under the control of the medical profession; second, Ohio and New York States. New York a few days only before Ohio, so Ohio ranks with the very first in dental legislation. Ohio does not rank with some of the other States of the Union, and we must say with some of the foreign countries, in dental legislation, while it should be in the foremost ranks in this matter.

Of course we can do nothing until our Legislature meets again, but meanwhile we can be making preparation, and I believe now that both those men who were interested in dental legislation, and those who were not, are all convinced that something must be done soon, and that we need it more than ever before. So we should have a committee representing all the various interests in the State, representing those out and in the societies, in the colleges and in practice, and they to prepare a bill which will be acceptable to all, and submit it to legal authority, so it will stand. Then, when the Legislature convenes, I think it will be very little trouble to have a bill of that kind passed, but if we continue to fight in factions, as we have before, we will have no success. It is true we are sometimes over-enthusiastic; if we believe we are right we will go ahead and let the results take care of themselves; it is not always best to state our convictions, very emphatically, for policy will win many times where straight up-and-down yes and no does not win. I believe the influences are such to-day that if it is properly handled we can get a bill which will be just.

"Why should we have a better law?" a prominent gentleman of another State said to me; "you are the dumping-ground for us." He said we ought to have legislation that would be similar to that of Pennsylvania and New York States. These two States wish to have us on an equal with themselves. We are not even on a par with the Western States. Michigan, for instance, will not accept everybody we do. It should not be so, we must have legislation in this State better than we have. The law in Pennsylvania requires all examination papers to be placed on file and kept for five years. If it is a question whether the examining board has done a creditable or discreditable act or not, the papers are open for inspection; if they stand for five years without being questioned, the evidence is conclusive, and the papers may be destroyed. The examination is under control of the Superintendent of Public Instruction. It should be so in our own State.

Our present laws require just a common school education for admittance to examination or to a college; the result of this is when a man graduates and goes out into society he has not the standing of a cultivated man.

The law, ministry and medicine will have a standing because of their educational acquirements. The dentist *per se* has not, but he may have, because he will make it in spite of his profession. The first requirement for entering a dental college should be to have a good literary preparation; that would be a step in advance. It would give him a standing in the community as a gentleman and a scholar.

Another thing is our dental board. While we now have a very excellent one, thanks to the governor, but who knows who will be on the next dental board, the next may possibly undo all the advance work we have at present. Now suppose we have the dental board examined, and they can be just as well as not. The dental boards should keep the colleges straight, and the colleges and societies should keep the boards filled with honorable men. We are all human beings, and personal interests will come in no matter how much we wish to have them out, they will have their influence. I believe it is the desire of nearly all of the college men, if they can, to throw a part of the responsibility on the general profession. The dental profession now throws all the responsibility on the college. The college should hold the commonwealth responsible for admitting a man to college. The college matriculate should have a better education than is now required.

The college instructor should not be required to work harder than any other man. I think the day is passed when a man can go into an office and receive the instruction necessary to make him an acceptable dentist, because there are so few men who are teachers, and the colleges should have the best men for instructors.

A very essential thing at present is to see that we have some means of punishing practitioners for malpractice; we have not adequate power at present. We should have some process which would make it impossible for an incompetent person to practice in this State.

Dr. Taft: Mr. President, I would inquire whether there is a Committee on Legislation or not. I think it is very important to have this matter in hand. There should be a committee which should be assigned to that duty, to see what change should be made in our present laws and have it ready for the next Legislature. If we let it go on to the nextmeeting, it will continue in that way and there will be nothing accomplished. If there is no such committee, one should be appointed now. I move, Mr. Chairman, that a Committe on Legislation, to consist of say five or as many as may be thought proper, be appointed to have this matter in hand, and the motion was seconded, presented and carried.

